# Effects of trunk extensor eccentric exercise on lipid profile and glycaemic response

**DOI:** 10.1136/bmjsem-2020-000861

**Published:** 2020-10-28

**Authors:** Ho-Seong Lee, Takayuki Akimoto, Ah-Ram Kim

**Affiliations:** 1 Department of Kinesiologic Medical Science, Dankook University - Cheonan Campus, Cheonan, Korea (the Republic of); 2 Institute of MEDI-Sports,Dankook University - Cheonan Campus, Cheonan, Korea (the Republic of); 3 Laboratory of Muscle Biology, Waseda University, Shinjuku-ku,Japan; 4 Physical Therapy, Namseoul University, Cheonan, Korea (the Republic of)

**Keywords:** Metabolism, Lipids, Glucose, Muscle damage/injuries

## Abstract

**Objectives:**

A number of previous studies reported physiological responses and adaptations after eccentric muscle contraction of limb muscles. In contrast, no study has determined physiological response after eccentric contraction of trunk muscles. The purpose of the present study was to compare the functional and metabolic changes after eccentric or concentric exercises of trunk extensor muscles.

**Methods:**

In this randomised, crossover study, 10 men performed a single bout of 50 maximal voluntary concentric and eccentric contractions of the trunk extensor with an interval of 2 weeks between bouts. The activities of the paraspinal muscles were recorded during concentric and eccentric contractions. Muscle soreness, muscle function, blood lipid profiles and glycaemic responses were measured before, immediately after and at 24, 48, 72 and 96 hours after each bout.

**Results:**

The lumbar multifidus and iliocostalis lumborum activities during eccentric contractions were significantly higher than those during concentric contractions (p<0.05). The maximal strength and muscle endurance of the trunk extensor were not decreased even after the eccentric contractions. Compared with concentric contractions, muscle soreness was significantly increased at 24, 48, 72 and 96 hours after eccentric contractions (p<0.05). The TG, TC and LDL-C were significantly lower at 48, 72 and 96 hours after eccentric contractions (p<0.05), while blood glucose levels and HOMA-IR were significantly greater at 48 and 72 hours after eccentric contractions (p<0.05).

**Conclusion:**

This study indicated that eccentric contractions of the trunk extensor had positive effects on the lipid profile and the glycaemic response.

## INTRODUCTION

Unaccustomed intense eccentric exercise causes muscle damage to induce muscle soreness, a reduction in strength and the release of intramuscular enzymes into blood.^1^ Structural damage to the muscle cells following eccentric exercise is thought to be caused by immediate mechanical trauma^2^ and subsequent chemically mediated processes.^3^ On the other hand, it is generally known that eccentric exercise brings greater benefits compared to concentric exercise including greater gains in muscle strength and mass.^4^ Specific physiological responses and adaptations to eccentric exercise of limb muscles have been reported in different tissues and organ systems. These include cardiovascular,^5^ respiratory,^6^ neuromuscular,^7^ endocrine^8^, bone,^9^ connective tissue,^10^ inflammatory,^11^ oxidative stress,^12^ lipid profile^13^ and insulin sensitivity^14^ responses.

Recent literatures have promoted the importance of core or trunk muscle strength for the successful performance of sports-related and everyday activities. Functionally, the trunk muscles play an important role as kinetic link that facilitates the transfer of torques and angular momenta between upper and lower extremities during the execution of whole-body movements as part of sports skills, occupational skills, fitness activities and activities of daily living^15^ Compared with many other limb muscles, trunk extensors are postural muscles rich in slow-twitch fibres,^16^ which have uncharacteristically larger diameters than fast-twitch fibres;^17^ they are responsible for slow and sustained contractions at relatively low force outputs. In spite of the important role of these muscles in performing activities of daily living, static/dynamic balance, mobility, injury prevention, there is no study investigating physiological responses of trunk muscles to eccentric exercise, so far.

The mechanical and metabolic properties of muscle fibres differ among fibre types and these characteristics of different muscles can vary greatly within the same individual. Evidence suggests that the variability in insulin-stimulated glucose uptake between different skeletal muscles may be partly due to differences in the muscle fibre composition and the expression of glucose transporter type 4 (GLUT-4), an insulin-responsive glucose transporter.^18^ Furthermore, it has been suggested that muscles with different fibre compositions may show different increases in insulin-independent glucose uptake following muscle contractions.

In addition, Lieber *et al* ^19^ suggested that the fast-twitch fibres might become fatigued at an early stage of the exercise period and might reach a state of rigour, depending on their ability to regenerate ATP. If muscle fibres in a state of rigour are subjected to subsequent stretching, damage may result. Alternatively, motor unit recruitment patterns may be responsible for the selective damage observed in specific fibre types, although glycogen depletion studies of eccentric exercise do not appear to support this.^20^

The aim of this study was to provide evidence that enhances our knowledge and understanding of physiological response of slow-twitch muscles to eccentric exercise, by investigating the functional and metabolic responses to eccentric exercise of the trunk extensor, which is mainly composed of slow fibres. On this basis, the present study was designed to compare the effects of eccentric versus concentric exercise of the trunk extensor on muscle function, blood lipid profiles and glycaemic response. We hypothesised that changes in the blood lipid and glycaemic profiles in response to eccentric exercise of the trunk extensor are different from these after concentric exercise, and that these changes would be significantly greater than those caused by concentric exercise.

## MATERIALS AND METHODS

### Subjects

Ten healthy men (age: 23.6±4.9 years, height: 176.9±2.5 cm, body mass: 74.9±7.3 kg, body mass index: 23.9±5.4 kg/m^2^) who had not been involved in a resistance-training programme for ≥6 months prior to the present study were enrolled; their characteristics are shown in table 1. The exclusion criteria were: (i) participation in any systematic training programmes at least 2 days per week, (ii) history of operation, severe trauma and/or fracture, (iii) presence of musculoskeletal diseases, (iv) any chronic diseases (eg, diabetes mellitus and epilepsy), (v) use of any medication that affects neuromuscular performance the last 2 weeks. The subjects reported no contraindication to exercise testing in a questionnaire. They were randomly assigned to perform a bout of maximal voluntary eccentric contractions or a bout of maximal voluntary concentric contractions in a cross-over fashion. The volunteers were asked to continue their usual routine (including their physical activity) without change throughout the study period. This study was approved by the Ethics Committee of Dankook University, Korea, in accordance with the ethical standards of the Declaration of Helsinki (IRB no. DKU 2019-09-035-001). They all provided written consent to participate in the study after being informed of the potential risks, discomforts and benefits involved.

**Table 1:** Changes in VAS-assessed muscle soreness and serum CK activity before (pre), immediately after (0) and 24, 48, 72 and 96 hours after eccentric and concentric contractions

Parameter	Contraction type	Pre	0 hour	24 hours	48 hours	72 hours	96 hours
VAS (mm)	ECC	0.0 ±0.0	14.3 ±3.1	52.8 ±10.4*	43.2 ±23.5*	38.7 ±16.7*	17.5 ±15.3*
	CON	0.0 ±0.0	12.1±2.4	0.0 ±0.0	0.0 ±0.0	0.0 ±0.0	0.0 ±0.0
CK activity (IU/L)	ECC	82.1 ±42.3	135.5 ±155.5	243.2 ±137.5	604.9 ±1125.5	427.6 ±379.7	401.0 ±412.9
	CON	68.4 ±53.7	89.8 ±43.1	89.9 ±43.1	76.1 ±29.6	68.6 ±38.5	81.4 ±46.6

*p<0.05 versus CON.

Values are means±SD.

CK, creatine kinase; CON, concentric; ECC, eccentric; VAS, visual analog scale.

### Study design

The study had a randomised, crossover design. Each subject performed bouts of concentric and eccentric exercise of the trunk extensor with an interval of 14 days between each bout. Both bouts of exercise were performed at the same time of day, and all measurements and blood samples were taken between 09:00 and 11:00 after an overnight fast and abstinence from alcohol and caffeine for 24 hours.^13^

**Figure 1:**
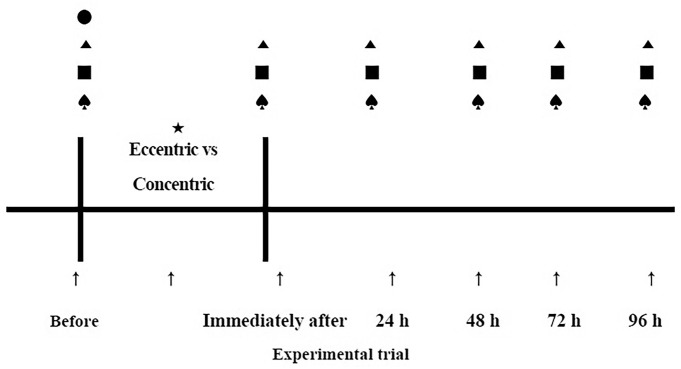
Schematic presentation of the experimental protocol. The figure shows the timing of physical characteristics (●), muscle soreness (▲), muscle function (■) and blood samples (♠) assessments. Muscle activity (★) was measured during each bout of exercise.

The changes in muscle function of the trunk extensor, blood lipid profile and glycaemic response were assessed before, immediately after and 24, 48, 72 and 96 hours after each bout of exercise (figure 1). Markers of muscle damage included subjective muscle soreness and serum creatine kinase (CK) activity. Surface electromyography (sEMG) of the hip, lumbar and thoracic trunk muscles was performed to evaluate muscle activity of the trunk extensor during the concentric and eccentric exercises. In each exercise bout, an electrode was placed to measure the maximal voluntary contraction (MVC) of each muscle and assess the performance of the concentric and eccentric exercises. All exercise and measurements were performed by same physical therapist.

### Exercise protocol

The exercises were performed on an isokinetic dynamometer (Cybex 6000; Lumex, Inc., Ronkonkoma, NY, USA) using HUMAC2004 software (Computer Sports Medicine, Inc., Stoughton, MA, USA). The subjects sat in a specially designed chair attached to the dynamometer. The chair stabilised the pelvis and lower extremities in order to isolate the centre of rotation of trunk movement to the L5–S1 motion segment. The L5–S1 interspace was palpated to align the axis of the actuator arm. The force output was sensed by a load cell attached to a hard rubber pad aligned with the inferior angle of the scapula.

Each subject completed a series of isometric contractions before performing each exercise protocol. As a warm-up and to familiarise themselves with the dynamometer, the subjects performed three 10-s submaximal (~50% MVC) isometric contractions of the trunk extensor muscles of 5 s each. After the warm-up contractions, maximal and submaximal test contractions, each preceded by 2 min of rest and lasting 5 s, were performed. The peak torque observed during one maximal contraction was recorded as 100% MVC. Next, the subjects performed isometric contractions at 20%, 40%, 60% and 80% MVC in a random order. Each submaximal torque level was maintained by the subject as they matched their real-time torque level to the objective torque level displayed on a computer monitor. All of the isometric contractions were performed with the subjects in an upright, neutral position (0°).^21^

The subjects then performed the exercise protocol, which consisted of a single bout of 50 eccentric or concentric contractions (at 100% MVC) of the trunk extensor muscles according to the assigned intervention. Eccentric contractions began with the trunk at 10° extension and ended at 40° flexion. Concentric contractions began with the trunk at 40° flexion and ended at 10° extension. Between the contractions, the subjects actively returned (without resistance) to the starting position. The movements were performed at an angular velocity of 20°·s^−1^.^22^ This angular velocity has been used before and was well tolerated by subjects performing maximal eccentric contractions using the trunk extensor muscles.^23^ One contraction was performed every 15 s.^22^

### Assessments

#### Muscle soreness

Muscle soreness was assessed on a 100-mm visual analog scale (VAS), where 0 mm indicated ‘no pain’ and 100 mm indicated ‘extremely painful’. The subjects were instructed to place a mark on the VAS while the trunk extensor was being forcibly flexed.

#### Muscle function

Muscle function was assessed by measuring the muscle strength and endurance of the trunk extensor. Muscle strength of the trunk extensor was measured based on the MVC. The MVC of the trunk extensors was assessed using the same apparatus as the exercise protocol.^22^ The procedures for these testing bouts were the same as those used before the exercise protocol, except that no warm-up was performed, as at least one submaximal contraction preceded the performance of the 100% MVC.

Muscle endurance of the trunk extensor was measured using the Sørensen test.^23^ The subjects lay in the prone position on the examining table, with the upper edge of the iliac crests positioned on the upper edge of the table. The pelvis, knees and ankles were fixed to the table by three straps, and the arms were bent. The subjects were asked to maintain their upper body in a horizontal position isometrically. A chair was placed in front of the subjects to help them support themselves by holding it with their hands in case they were unable to keep the position. The length of time during which the subjects kept their upper body straight and horizontal was recorded with a chronometer.

#### Blood sample

The serum levels of triacylglycerol (TG), total cholesterol (TC) and high-density lipoprotein cholesterol (HDL-C) were assayed by enzymic spectrophotometric methods using a CHOL kit, a TG kit and a HDL-C plus 3rd generation kit on a Modular Analytics system (all Roche, Mannheim, Germany). These biochemical parameters were determined in duplicate, simultaneously with a control serum from Roche. LDL-C was calculated using the equation: LDL-C = TC − HDL-C − (TG/5), as previously described.^16^ The TC/HDL-C ratio was also calculated as an atherogenic index.

Serum glucose was assayed using an enzymatic kinetic assay (hexokinase) (glu kit; Roche Diagnostics, Mannheim, Germany). Serum insulin was determined using an electrochemiluminescence immunoassay (insulin kit; Roche Diagnostics, Mannheim, Germany). Homoeostasis model assessment (HOMA-IR) was used as a surrogate measure of insulin resistance, and was calculated as fasting insulin (µU·mL^−1^)×fasting glucose (mmol·mL^−1^)/22.5. Whole-blood glycosylated haemoglobin (HbA1c) was measured using an HbA1c kit on a NycoCard Reader II (both Axis-Shield, Dundee, Scotland).^14^ Creatine kinase (CK) was measured using a Reflotron spectrophotometer kit (Boehringer-Manheim, Pode, Czeck Republic).

#### Electromyography

The sEMG signals of seven unilateral muscles were measured with an eight-channel sEMG system (Trigno Wireless EMG System, Delsys Inc., Boston, MA, USA). To reduce skin impedance and improve skin contact, the skin was prepared by shaving and rubbed with alcohol. After skin preparation, surface electrodes were bilaterally attached parallel to the muscle fibre orientation over the following muscles^23 24^: gluteus maximus (GM—midway between the posterosuperior iliac spine and the ischial tuberosity), the lumbar multifidus (LM—2 cm lateral to the midline of the body, above and below a line connecting the posterior and superior iliac spines), the latissimus dorsi (LD—3 cm lateral and caudal to the angulus inferior of the scapula), the longissimus thoracis pars thoracic (LTT—at the L1 level, midway between the line through the spinous process and a vertical line through the posterosuperior iliac spine), the longissimus thoracis pars lumborum (LTL—lateral at the intersection of a horizontal line through the spinous process of L5 and a line between the interspinous space of L1-L2 and the posterosuperior iliac spine), the iliocostalis lumborum pars thoracis (ILT—at the L1 level, midway between the lateral palpable border of the erector spinae and a vertical line through the posterosuperior iliac spine) and the iliocostalis lumborum pars lumborum (ILL—at the L4 level, midway between the lateral palpable border of the erector spinae and a vertical line through the posterosuperior iliac spine). Muscle activities were measured during all phases of the contraction exercise.

### Statistical analyses

The Shapiro–Wilk test was used to confirm that all of the dependent variables were normally distributed. The effects of the eccentric and concentric contraction types on the dependent variables during the exercise protocol were evaluated by two-way analysis of variance (time × contraction) with repeated time measures. When a significant time × contraction interaction effect was observed, Tukey’s post hoc test was performed to compare the eccentric and concentric contractions at each time-point separately. Values of p<0.05 were considered statistically significant. The results are presented as means±SD. All analyses were conducted using SPSS software version 21.0 (SPSS Inc., Chicago, IL, USA).

## RESULTS

### Markers of muscle damage

The changes in muscle soreness and serum CK activity are shown in table 1. Eccentric contractions had significantly higher muscle soreness at 24, 48, 72 and 96 hours post-exercise as compared with concentric contractions (p<0.05). No significant changes in CK activity observed after either contraction type. However, owing to the large intra-subject variability in CK levels, we found no significant changes in serum CK activity after eccentric contractions. Nevertheless, all subjects showed increases in CK levels from baseline following eccentric contractions.

### Muscle function

**Figure 2:**
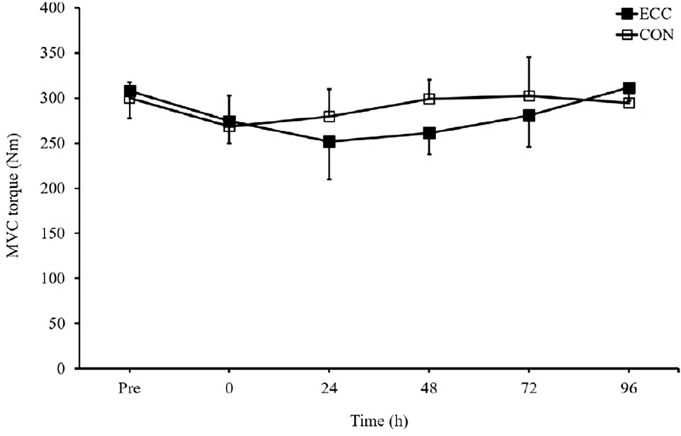
Maximal voluntary contraction measured before (pre), immediately after (0) and 24, 48, 72 and 96 hours after concentric and eccentric contractions. Values are means ± SD. CON, concentric; ECC, eccentric; MVC, maximal voluntary contraction.

**Figure 3:**
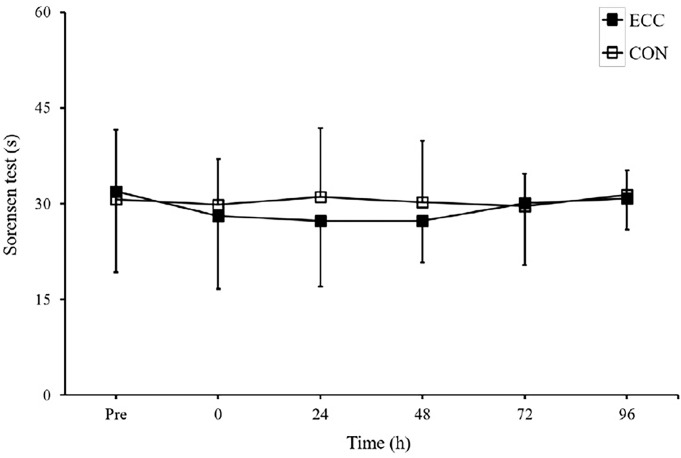
Results of the Sørensen test performed before (pre), immediately after (0) and 24, 48, 72 and 96 hours after eccentric and concentric contractions. Values are means ± SD. CON, concentric; ECC, eccentric.

The changes in MVC torque and the results of the Sørensen test are shown in figures 2 and 3. MVC torque was not significantly different in the contraction type generating ability at any time-point. Moreover, the Sørensen test showed no significant difference in muscle endurance at any time-point following concentric contractions.

### Lipid profiles and glycaemic responses

The changes in blood lipid profiles and glycaemic responses are shown in table 2. Compared with concentric contractions, eccentric contractions had significantly lower TG levels at 24, 48, 72 and 96 hours post-exercise, together with lower TC, LDL-C and TC/HDL-C at 48, 72 and 96 hours post-exercise (p<0.05). By contrast, no significant changes in HDL-C levels were observed after either contraction type. Eccentric contractions had significantly higher glucose levels (p<0.01) and HOMA-IR (p<0.05) compared with concentric contractions at 48 and 72 hours post-exercise. By contrast, no significant changes in insulin or HbA1c were observed after either contraction type.

**Table 2:** Changes in lipid profiles and glycaemic responses before (pre), immediately after (0) and 24, 48, 72 and 96 hours after eccentric and concentric contractions

Parameter	Contraction type	Pre	0 hour	24 hours	48 hours	72 hours	96 hours
TG (mM)	ECC	0.82 ±0.10	0.71 ±0.13	0.67 ±0.07*	0.69 ±0.12*	0.71 ±0.04*	0.73 ±0.13*
	CON	0.83 ±0.12	0.81 ±0.11	0.82 ±0.09	0.83 ±0.18	0.82 ±0.10	0.84 ±0.08
TC (mM)	ECC	4.42 ±0.43	4.35 ±0.33	4.01 ±0.75	3.79 ±0.51*	3.82 ±0.97*	3.91 ±0.92*
	CON	4.48 ±0.53	4.46 ±0.97	4.40 ±1.03	4.45 ±0.93	4.50 ±0.71	4.49 ±0.97
HDL-C (mM)	ECC	1.37 ±0.19	1.35 ±0.11	1.49 ±0.17	1.52 ±0.21	1.49 ±0.18	1.39 ±0.15
	CON	1.41 ±0.23	1.44 ±0.17	1.46 ±0.24	1.44 ±0.19	1.43 ±0.21	1.42 ±0.16
LDL-C (mM)	ECC	2.78 ±0.36	2.81 ±0.33	2.58 ±0.28	2.21 ±0.29*	2.13 ±0.21*	2.24 ±0.32*
	CON	2.71 ±0.34	2.74 ±0.28	2.56 ±0.37	2.64 ±0.29	2.63 ±0.34	2.72 ±0.36
TC/HDL-C	ECC	3.23 ±0.23	3.22 ±0.30	2.69 ±0.44	2.49 ±0.24*	2.56 ±0.54*	2.81 ±0.61*
	CON	3.18 ±0.23	3.10 ±0.57	3.01 ±0.43	3.09 ±0.49	3.15 ±0.34	3.16 ±0.60
Glucose (mmol/L)	ECC	4.44 ±0.16	4.58 ±0.23	4.83 ±0.11	4.96 ±0.12*	4.91 ±0.11*	4.76 ±0.08
	CON	4.55 ±0.21	4.52 ±0.16	4.63 ±0.29	4.61 ±0.24	4.57 ±0.13	4.58 ±0.23
Insulin (µU/mL)	ECC	13.10 ±1.78	13.92 ±2.03	14.87 ±1.39	15.40 ±2.11	14.91 ±1.21	14.48 ±2.37
	CON	13.10 ±2.14	13.27 ±3.12	13.03 ±1.78	12.80 ±2.48	13.02 ±1.62	13.15 ±1.93
HOMA-IR	ECC	2.60 ±0.41	2.71 ±0.27	2.92 ±0.47	3.18 ±0.34*	3.07 ±0.29*	2.84 ±0.28
	CON	2.68 ±0.74	2.71 ±0.52	2.65 ±0.39	2.66 ±0.69	2.67 ±0.41	2.69 ±0.24
Glycosylated haemoglobin (%)	ECC	5.58 ±0.14	5.56 ±0.29	5.57 ±0.33	5.55 ±0.11	5.54 ±0.17	5.57 ±0.27
	CON	5.59 ±0.07	5.58 ±0.13	5.52 ±0.14	5.49 ±0.10	5.50 ±0.17	5.53 ±0.21

*p<0.05 versus CON.

Values are means±SD.

CON, concentric; ECC, eccentric; HDL-C, high-density lipoprotein cholesterol; HOMA, homoeostasis model assessment index; LDL-C, low-density lipoprotein cholesterol; TG, triacylglycerol; TC, total cholesterol.

### Electromyography

**Figure 4:**
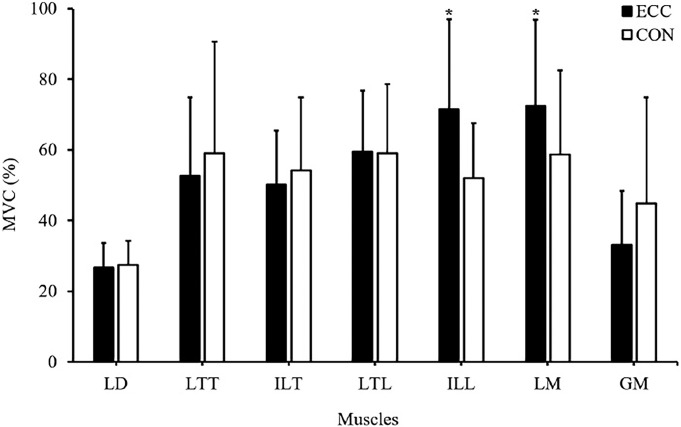
Activities of paraspinal muscles during concentric and eccentric contractions. Muscle activities are presented as the per cent maximal voluntary contraction following each contraction type. Values are means ± SD. * p<0.05 versus CON. CON, concentric; ECC, eccentric; GM, gluteus maximus; LD, latissimus dorsi; ILL, iliocostalis lumborum pars lumborum; ILT, iliocostalis lumborum pars thoracic; LM, lumbar multifidus; LTL, longissimus thoracis pars lumborum; LTT, longissimus thoracis pars thoracic.

The changes in muscle activity are shown in figure 4. LM activity was significantly greater during eccentric contractions than during concentric contractions (p<0.05), as was ILL activity (p<0.05). By contrast, there were no significant differences in muscle activities in the LD, LTT, ILT, LTL and GM between eccentric and concentric contractions.

## DISCUSSION

The results of this study show that a single bout of maximal eccentric contractions of the trunk extensor muscles induced different post-exercise lipid and glycaemic responses from concentric exercise. Nevertheless, there were increases in LM and ILL activities during eccentric contractions as compared with during concentric contractions, and subjects reported greater muscle soreness between 24 and 72 hours following eccentric contractions. However, no changes in muscle function were observed after eccentric and concentric contractions.

In limb muscles, a reduction in isometric force generation lasting for several days after a single bout of eccentric contractions was observed in studies of human knee extensor muscles^25^ and elbow flexor muscles.^3 26^ In one study, a decrease in force-generating ability persisted for >2 weeks after an eccentric exercise bout.^27^ Sustained increases in muscle activity required to elicit a given submaximal force were also observed after a bout of eccentric contractions.^26 27^ Unlike these earlier studies, the present study did not detect any sustained changes in isometric torque generation or muscle activity after eccentric contraction. One explanation for this may be due to the difference in fibre type composition between the lumbar paraspinal muscles and limb muscles. It is generally accepted that type II fibres are preferentially damaged by eccentric contractions.^28^ The lumbar paraspinal muscles have a high percentage (~60%) of type I fibres,^17^ which may render them less susceptible to eccentric exercise-induced muscle damage.

Another possible explanation may be due to the nature of the lumbar paraspinal muscles. Because these muscles are important in maintaining an upright posture, they are more chronically active and may be ‘trained’ such that a bout of maximal eccentric contractions does not elicit sustained changes in torque production or muscle activity, as compared with the effects on limb muscles reported in prior studies. In support of this, there is evidence that recent contractile history is a better determinant of susceptibility to eccentric exercise-induced muscle damage than the fibre type composition.^29^

TG levels remained diminished for 96 hours after the eccentric exercise. Increased lipoprotein lipase activity may be related to the increased demand of working muscles for fatty acids as an energy source, and to replenish muscle phospholipid and TG stores with fatty acids to regenerate damaged muscle fibres.^30^ The lower serum TG levels after muscle-damaging exercise may also be due to increases in resting energy expenditure that last several days after eccentric exercise,^31^ corresponding to an increased need for ATP, mainly for the regeneration of damaged fibres and/or for the formation of new muscle fibres from satellite cells. Because cholesterol constitutes approximately 13% of cell membrane^32^ and signs of healing have been observed in human subjects within 36 hours after eccentric exercise^2^, it is possible that the reductions in serum TC and LDL-C levels after eccentric contractions were due to outflow of cholesterol from plasma to muscle to facilitate synthesis of new cell membrane. However, it is shown that normal range of TC and LDL-C may be due in part to the lower levels of muscle damage experienced, because less cholesterol molecules would be needed for the repair process that takes place in the damaged muscle cells.

Many studies have reported that acute eccentric exercise in limb muscles increases insulin resistance and plasma insulin levels, and decreases glucose disposal rates.^14^ Eccentric exercise has been shown to cause muscle damage and impaired post exercise glycogen resynthesis^33^ and causes whole-body insulin resistance,^34^ but no underlying mechanisms have been established. It has been suggested that accumulation of inflammatory cells after eccentric exercise may be the cause of impaired glycogen resynthesis due to competition between the inflammatory cells and muscle fibres for available plasma glucose.^35^ Furthermore, inflammatory cells have been shown to produce a factor that stimulates glycolytic flux in muscle,^36^ thus possibly diverting glucose away from muscle glycogen synthesis. Indeed, the present study found increased plasma glucose levels and HOMA-IR after eccentric exercise of the trunk extensor.

Although the lumbar and thoracic paraspinal muscles can act synergistically to produce an extension force, several studies have suggested that the back muscles are not allied muscle fibres,^23^ but are composed of different groups of fascicles with different functions. Therefore, it is necessary to differentiate between the thoracic and lumbar muscle groups based on their anatomical and functional differences. Although both muscle groups cross the lumbar spine, the lumbar muscle parts directly attach to the lumbar vertebrae, while the thoracic parts originate from the thorax and insert themselves in long tendons that form the erector spinae aponeurosis.^37^ The thoracic muscles, which are more superficial, are primarily force-producing muscles whereas the deeper lumbar muscles (especially the LM) are primarily stabilising muscles in the spine. In this study, the activities of the LM and ILL were greater during eccentric contractions than during concentric contractions. The LM has been shown to make a major contribution to the control and segmental stabilisation of the lumbar spine,^21^ whereas the ILL clearly has a torque-producing and general trunk stabilising function.^24^ Furthermore, the ILL is only recruited at higher force levels during trunk extension.^38^ It seems that there is need to more stabilisation and torque-producing during eccentric contractions than during concentric contractions.

## CONCLUSION

In conclusion, this study showed that eccentric exercise of the trunk extensor induced different changes in blood lipid and glycaemic profiles from these after concentric exercise. Further studies are needed to verify and extend the present findings, and to investigate the effects of repeated bouts of eccentric exercise of the trunk extensor on blood lipid profiles and glycaemic responses.

**Summary box**

**What are the new findings?**

- A single bout of maximal eccentric contractions of the trunk extensor muscles had positive effects on the lipid profile and the glycaemic response compare to concentric contractions.
- LM and ILL activities increases during eccentric contractions as compared with during concentric contractions.
- No changes in muscle function were observed after eccentric and concentric contractions of the trunk extensor muscles.

**How might it impact on clinical practice in the future?**

- Awareness of the potential metabolic changes following eccentric exercise may help coaches, exercise scientists and health and fitness practitioners to make more informed decisions about the advice they give to different people.
- A better insight into the mechanisms governing eccentric exercise adaptations should provide invaluable information for designing therapeutic interventions and identifying potential therapeutic targets.
